# Effect of Adding Resistant Maltodextrin to Pasteurized Orange Juice on Bioactive Compounds and Their Bioaccessibility

**DOI:** 10.3390/foods10061198

**Published:** 2021-05-26

**Authors:** Elías Arilla, Purificación García-Segovia, Javier Martínez-Monzó, Pilar Codoñer-Franch, Marta Igual

**Affiliations:** 1 Food Investigation and Innovation Group, Food Technology Department, Universitat Politècnica de València, Camino de Vera s/n, 46022 València, Spain; elarco@upv.es (E.A.); xmartine@tal.upv.es (J.M.-M.); marigra@upvnet.upv.es (M.I.); 2 Department of Pediatrics, Obstetrics and Gynecology, University of València, Avenida de Blasco Ibáñez, No. 15, 46010 València, Spain; pilar.codoner@uv.es; 3 Department of Pediatrics, University Hospital Dr. Peset, Foundation for the Promotion of Health and Biomedical Research un the Valencian Region (FISABIO), Avenida Gaspar Aguilar, No. 90, 46017 València, Spain

**Keywords:** resistant maltodextrin, orange pasteurized juice, bioactive compounds, bioaccessibility

## Abstract

Resistant maltodextrin (RMD) is a water-soluble and fermentable functional fiber. RMD is a satiating prebiotic, reducer of glucose and triglycerides in the blood, and promoter of good gut health, and its addition to food is increasingly frequent. Therefore, it is necessary to study its potential effects on intrinsic bioactive compounds of food and their bioaccessibility. The aim of this study was to evaluate the effect of adding RMD on the bioactive compounds of pasteurized orange juice with and without pulp, and the bioaccessibility of such compounds. RMD was added at different concentrations: 0 (control sample), 2.5%, 5%, and 7.5%. Ascorbic acid (AA) and vitamin C were analyzed using HPLC, whereas total phenols, total carotenoids (TC), and antioxidant capacity were measured using spectrophotometry. After that, sample in vitro digestibility was assessed using the standardized static in vitro digestion method. The control orange juice with pulp presented significantly higher values of bioactive compounds and antioxidant capacity than the control orange juice without pulp (*p* < 0.05). RMD addition before the juice pasteurization process significantly protected all bioactive compounds, namely total phenols, TC, AA, and vitamin C, as well as the antioxidant capacity (AC) (*p* < 0.05). Moreover, this bioactive compound protective effect was higher when higher RMD concentrations were added. However, RMD addition improved phenols and vitamin C bioaccessibility but decreased TC and AA bioaccessibility. Therefore, the AC value of samples after gastrointestinal digestion was slightly decreased by RMD addition. Moreover, orange pulp presence decreased total phenols and TC bioaccessibility but increased AA and vitamin C bioaccessibility.

## 1. Introduction

Fruit consumption has historically been associated with a healthy diet, as they provide key nutrients, especially bioactive compounds such as vitamins, minerals, dietary fiber, and phytochemicals. They have also been linked to a reduced risk of developing chronic diseases such as cardiovascular disease, cancer, diabetes, or aged-related functional decline [1]. Furthermore, in the last decades, consumers have developed a greater awareness of how food affects their health and wellbeing. This, along with the consumers’ need to save time, is causing the fruit juice sector to experience a positive and dynamic growth. Thus, fruit juices are becoming part of the so-called new-age beverages [2].

With this pathway to a better diet, the juice industry has focused on product differentiation and development of juices that go beyond basic nutrition and good tasting [3,4]. This new paradigm expanded the limits of an already mature market, introducing novel functional ingredients that are founded on the premise that, beyond its nutritional contribution, helps to promote optimal health conditions and reduce the risk of diseases through positively modulating host gut microbiota [5,6]. One of these novel functional ingredients are prebiotics, which are defined as ”selectively fermented ingredients that allow specific changes, both in the composition and/or activity in the gastrointestinal microflora that confers benefits upon host wellbeing and health” [7]. Prebiotics can improve the survival, growth, metabolism, and beneficial health activities of probiotics in the digestive system [5]. Many oligosaccharides and polysaccharides–including dietary fiber–have been studied because of their potential prebiotic activity, such as inulin, fructo-oligosaccharides, lactulose, isomalto-oligosaccharides, lactosucrose, xylo-oligosaccharides, gluco-oligosaccharides, and human milk oligosaccharides, among others [7,8]. Resistant maltodextrin (RMD) is a dietary fiber that has attracted a lot of interest in recent times. RMD is a water-soluble fiber produced from the heat treatment of corn starch, indigestible in the small intestine but possibly fermented in the colon, resulting in enhanced short-chain fatty acid production [9]. It has been proven to exert a satiating effect [10], to reduce post-meal glucose [11] and triglycerides [12] levels in blood, and to promote good gut health [13].

Fruit juices have been suggested as an ideal medium for such functional ingredients because of their natural content of beneficial nutrients, and because they are generally well-accepted by all age groups in terms of organoleptic properties [14,15]. Among all fruit juices, the most valued is orange juice, which was the most consumed fruit juice worldwide in 2018, representing 43.8% of the fruit juice market [16]. Moreover, orange juice provides an important dietary source of bioactive compounds, such as phenolic compounds, carotenoids, ascorbic acid (AA), and vitamin C [17], that contribute to its antioxidant properties. Since bioactive compounds are associated with better diet quality and an increase of positive health outcomes [18], it is important to optimize their stabilization in the food matrix, especially in those food products subjected to industrial processes.

Pasteurization is the most widely used preservation method for fruit juices, as it is the most cost-effective method [19]. However, although thermal processing gives microbial safety, it causes irreversible losses of bioactive compounds and antioxidant properties [20,21], thus reducing their beneficial health effects. Therefore, it is important to evaluate whether adding a prebiotic fiber, such as RMD, positively or negatively affects the preservation of intrinsic bioactive compounds in pasteurized orange juice. Furthermore, it is important to explore the food processing field of RMD addition to elucidate its effects on the intrinsic bioactive compounds of orange juice. It is also necessary to analyze whether incorporating functional ingredients plays a role in the bioaccessibility of health-promoting compounds in the gastrointestinal tract, since bioaccessibility represents compounds that may be absorbed in the gut [22]. Therefore, in vitro models provide information on the pre-absorption phase that can be expressed quantitatively based on the fraction of bioactive compounds released from the food matrix during ingestion [23]. This analyses if the desired functionality is achieved through improving the protection and release of such compounds from the food matrix.

Most studies have mainly focused on the effect of prebiotic addition on stability, storage conditions, or functionality of the prebiotic fiber in the finished beverage [24,25,26,27]. Therefore, it is necessary to evaluate the addition of a potential prebiotic fiber such as RMD and its impact on the food matrix. Furthermore, the effect of RMD addition to the physico-chemical properties of pasteurized orange juice was studied [28].

This study aimed to evaluate the effect of RMD addition before pasteurization treatment on the bioactive compounds (total phenols, total carotenoids (TC), AA, and vitamin C) and the antioxidant capacity (AC) of orange juice. Moreover, an in vitro digestion simulation was performed to analyze their bioaccessibility.

## 2. Materials and Methods

### 2.1. Raw Materials

This study was conducted with freshly squeezed orange juice supplied by Refresco Iberia S.A.U. (Valencia, Spain). All oranges were from Spanish origin. RMD (Fibersol-2) added to the juice was purchased from ADM/Matsutani, LLC (Decatur, IL, USA). Frozen pasteurized orange pulp was provided by a local fruit processing company (Zumos Valencianos del Mediterráneo, Valencia, Spain).

### 2.2. Sample Preparation and Pasteurization

Eight orange juice samples were prepared to conduct this study. Four were with orange pulp-added (orange juice with pulp, OJP), and four were without orange pulp (orange juice without pulp, OJWP). In order to minimize differences between samples, one single batch of orange juice was selected and separated into two tanks. Orange pulp was added to one tank and the other was left pulp-free. Pulp content was homogenized using a stirrer (LH Overhead Stirrer, VELP Scientifica, Usmate, Italy), by applying 200 rpm for 5 min. Then, each tank was transferred to 4 new tanks (8 in total) to add increasing RMD concentrations (2.5%, 5%, and 7.5%). This way, for a finished drink portion of 200 g, 5, 10, and 15 g of RMD would be ingested, enough to display its prebiotic effect. Control samples without RMD were also prepared. All orange juices were pasteurized (Fruchtsaftdispenser, Mabo Steuerungselement GmbH, Eppingen, Germany) at 85 °C for 10 s and hot-filled in 250 mL polyethylene terephthalate (PET) bottles.

### 2.3. Analytical Determinations

#### 2.3.1. Brix, Acidity, and pH

Measurement of total soluble solids (TSS) by refractometry (Abbemat 200, Anton Paar, Austria), acidity as grams of citric acid per 100 mL (g AC/100 mL) (DL53 acid titrator, Mettler Toledo, Switzerland), and pH (Basic 20 pH meter, Crison, Spain) were performed as basic quality control parameters for the orange juices.

#### 2.3.2. Total Phenols (TP)

Determining TP was based on the Folin–Ciocalteu method. The extraction procedure consisted of homogenizing 35 g of the orange juice for 1 min with 50 mL of methanol. The homogenate was centrifuged (10,000 rpm, 10 min, 4 °C) to obtain the supernatant. Then, 15 mL of distilled water and 1.25 mL of Folin–Ciocalteu reagent (Sigma-Aldrich, Steinheim, Germany) were added to 250 µL of the supernatant. The samples were mixed and allowed to stand for 8 min in darkness before 3.75 mL of 7.5% sodium carbonate aqueous solution was added. Water was added to adjust the final volume to 25 mL. Samples were allowed to stand for 2 h at room temperature before measurement. Absorbance was measured at 765 mm in a UV-visible spectrophotometer (Thermo Electron Corporation, Waltham, MA, USA). The total phenolic content was expressed as mg of gallic acid equivalents (GAE) (Sigma-Aldrich, Steinheim, Germany) per 100 mL or g of orange juice [29]. Samples were analyzed in triplicate before and after in vitro digestion.

#### 2.3.3. Total Carotenoids (TC)

The TC in the samples before and after in vitro digestion were extracted with a solvent hexane/acetone/ethanol mixture following the method of Olives et al. [30] in triplicate. Sample absorbance was measured at 446 nm in a UV-visible spectrophotometer (Thermo Electron Corporation). The TC content was expressed as mg of β-carotene (Fluka-Biochemika) per 100 mL or g of orange juice.

#### 2.3.4. Ascorbic Acid (AA) and Vitamin C

AA and vitamin C (ascorbic acid + dehydroascorbic acid) were determined using a HPLC-UV detector (Jasco equipment, Italy) in triplicate. The method proposed by Xu et al. [31] was used to determine the ascorbic acid with some modifications made by Igual et al. [29]. To determine the ascorbic acid, 1 g of the sample was extracted with 9 mL 0.1% oxalic acid for 3 min and immediately filtered (0.45 µm) before injection. The procedure employed to determine total vitamin C was the reduction of dehydroascorbic acid to ascorbic acid, using DL-dithiothreitol as the reductant reagent. A 0.5 mL aliquot sample was taken to react with 2 mL of a 20 g/L dithiothreitol solution for 2 h at room temperature and in darkness. Afterwards, the same procedure as that used for the ascorbic acid method was performed. The HPLC method and instrumentation was Ultrabase-C18, 5 µm (4.6 × 250 mm) column (Scharlab, Barcelona, Spain); mobile phase 0.1% oxalic acid, volume injection 20 µL, flow rate 1 mL/min, detection at 243 nm and at 25 °C. An AA standard solution (Sigma-Aldrich, Steinheim, Germany) was prepared.

#### 2.3.5. Antioxidant Capacity (AC)

AC was assessed using the free radical scavenging activity of the samples evaluated with the stable radical 2,2-diphenyl-1-picryl-hydrazyl-hydrate (DPPH) following the methodology of Igual et al. [32] in triplicate. Samples were mixed with methanol. The homogenate was centrifuged (10,000 rpm, 10 min, 4 °C) to obtain the supernatant. A total of 0.1 mL of supernatant was added to 3.9 mL of DPPH (0.030 g/L, Sigma-Aldrich, Steinheim, Germany) in methanol. A UV-visible spectrophotometer (Thermo Electron Corporation) was used at the absorbance of 515 nm. The results were expressed as milligram Trolox equivalents (TE) per 100 mL or g.

### 2.4. In Vitro Digestion

Sample in vitro digestibility (IVD) (%) was assessed using the standardized static in vitro digestion method suitable for food (COST INFOGEST network) proposed by Minekus et al. [33]. Four steps were followed: the oral phase, mixing the sample and simulate salivary fluid (SSF) (1:1) with amylase at pH 7 for 2 min; the gastric phase, mixing the oral bolus and simulate gastric fluid (SGF) (1:1) with pepsin at pH 3 for 2 h; the intestinal phase, mixing the gastric chyme and simulate intestinal fluid (SIF) (1:1) with enzymes at pH 7 for 2 h; centrifuging at 4500 rpm for 30 min and then a filtration, filtering through a 1 µm glass-fiber membrane [34].

The in vitro digestibility was calculated as the difference between the initial mass and the undigested mass (after correcting for the blank assay), divided by the initial mass, and multiplied by 100, according to Batista et al. [35]. Analyses were repeated in triplicate. Samples obtained at the end of in vitro digestion were collected according to Minekus et al. [33]. These were freeze-dried with a protease inhibitor. With crude protein values calculated, the bioaccessibility was determined using Equation (1), proposed by Khouzam et al. [36].

(1) $$\mathrm{Bioaccessibility}=\left(\frac{A}{B}\right)\times 100,$$

where, A is the concentration of the bioactive compounds in the bio-accessible fraction after in vitro digestion (filtrate after filtration); B is the concentration of the bioactive compounds in the sample before digestion. Possible bioactive compounds present in tap water and the reagents were also analyzed and corrected in the final bio-accessible fraction.

### 2.5. Statistical Analysis

Analysis of variance (ANOVA) was applied with a confidence level of 95% (*p* < 0.05), to evaluate the differences between the samples. Furthermore, a correlation analysis among studied bioactive compounds and antioxidant capacity of juices, with a 95% significance level was conducted. Statgraphics Centurion XVII Software, version 17.2.04 (Statgraphics Technologies, Inc., The Plains, VA, USA) was used.

## 3. Results and Discussion

### 3.1. Effect of RMD on TSS, Acidity, pH, and Bioactive Compounds of Pasteurized Orange Juice

TSS, acidity, and pH (Table 1) were evaluated as basic quality control parameters, as they are related to the stability of bioactive compounds in plant-derived products [17]. Control samples without RMD (OJP0 and OJWP0) agreed with values obtained by other authors [17,37,38]. In orange juice, soluble solids are sugars, mainly fructose, sucrose, and glucose, that, with the citric acid content, determine the characteristic balance of sweetness and sourness that makes orange juice appealing to consumers [37]. Increasing RMD concentrations led to increasing TSS values (*p* < 0.05). RMD is a water-soluble fiber and, therefore, dissolves in aqueous matrices, such as orange juice. Other studies have also reported that prebiotic fiber addition led to an increase in TSS in fruit-based beverages [32,39]. Therefore, prebiotics go beyond their functional properties contributing to sweetness, texture, and mouthfeel and have been proposed as sugar replacers [40,41]. Nevertheless, OJP samples had slightly lower soluble solids content because they contained orange pulp, which is an insoluble fiber.

Contrary to the TSS, citric acid content significantly decreased (*p* < 0.05) with higher RMD concentrations in both OJP and OJWP samples, because its addition implied the replacement of raw orange juice in the finished beverage. Moreover, orange pulp addition seemed to play a role in the citric acid content, because OJWP had significantly lower acidity values (*p* < 0.05) than OJP samples. This suggests that orange pulp contains higher citric acid content than orange juice; therefore, its addition increased citric acid values. It also played a significant role (*p* < 0.05) in terms of pH. This similar behavior for TSS, acidity, and pH were reported in our previous paper on the same topic [28].

Oxidative stress is related to several detrimental effects on human health [42,43]. To manage this condition through diet, maintaining the maximum amount of antioxidant compounds from the food matrix is key, as antioxidants may prevent or delay oxidative cell damage [44]. Citrus fruits, and especially orange juice, have been reported to provide an important dietary source of bioactive compounds, including TP, TC, AA, and vitamin C, which contribute to its AC [17,45]. Table 2 compiles the mean values (with standard deviations) of these compounds and AC of control OJP0 and OJWP0 samples. OJP0 showed higher (*p* < 0.05) TP content than OJWP0, meaning that pulp addition to orange juice may increase TP content in the finished beverage. This may be because orange pulp contains hesperidin, which is the main phenolic compound in oranges for health-promoting activities [46]. Sádecká et al. [45] also reported that pulp-added pasteurized orange juice showed higher TP content (hesperidin) compared to pasteurized OJWP. Moreover, De Ancos et al. [47] found that orange pulp had a 1.6 times higher TP concentration than orange juice. In addition, OJP0 also obtained significantly higher (*p* < 0.05) TC content than OJWP0. Other studies on citrus products also demonstrated the importance of pulp in terms of TC content. For example, Rodrigo et al. [48] found an increase close to 40% in the TC content in orange pulp compared to freshly prepared orange juice. Following the same trend as with TP and TC content, OJP0 exhibited higher (*p* < 0.05) AA and vitamin C contents than OJWP0. This could be explained by the fact that citrus by-products, including orange pulp, have been suggested as a good source of AA in terms of quantity [49]. Thus, OJP0 had higher AC than OJWP0 (*p* < 0.05), exclusively because of orange pulp presence. Therefore, orange pulp addition to orange juice could be beneficial, leading to an increased AC.

Some differences were found between the bioactive compounds registered in both OJP0 and OJWP0 samples compared to other orange and citrus juice-based studies. Agcam et al. [21] showed lower TP content in orange juices treated using pulsed electric fields and conventional pasteurization. Moreover, Stinco et al. [50] and Velázquez Estrada et al. [51] reported lower TC content in fresh, high-pressure homogenized and pasteurized orange juices. Similarly, Sánchez-Moreno et al. [17] also had a lower TC content in commercial orange juices stored at room, cold, and frozen temperatures. In contrast, Aschoff et al. [52] found higher AA and vitamin C contents in fresh and pasteurized orange juices. Bioactive compounds content in fruit-based juices is influenced by many aspects, such as the post-harvest, cultivar, or processing system. Therefore, it may be difficult to find similar ranges of bioactive compounds, as many aspects influence its quantification.

To evaluate the effect of RMD addition on the intrinsic bioactive compounds of pasteurized orange juice, the variation of each component (Δ*M_i_*) in all RMD-added samples with increasing RMD concentrations (2.5%, 5%, and 7.5%), referred to as OJP0 and OJWP0, respectively, was calculated according to Equation (2):

(2) $$\Delta M_{i}^{\mathrm{RMD}\%}=\frac{\left(M_{i}^{\mathrm{RMD}\%}-M_{i}^{\mathrm{Control}}\right)}{M_{i}^{\mathrm{Control}}}\times 100,$$

where, *M_i_*: mass of compound *i* in the sample obtained from 100 g of pasteurized orange juice control (OJP and OJWP) and superscripts, RMD%: percentage of RMD of the sample (2.5, 5, and 7.5) and control (OJP or OJWP).

According to this equation, the greater the positive variation of each pasteurized orange juice RMD-added sample compared to its control sample, the greater the protective effect RMD displays on the intrinsic bioactive compounds of orange juice, as higher amounts of each bioactive compound would be found.

**Figure 1:**
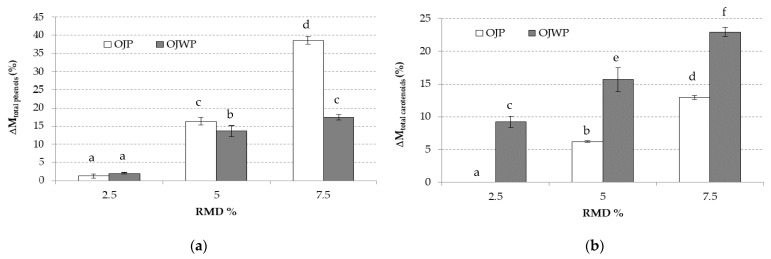
(**a**) Mean values and standard deviation of total phenols variation of pasteurized orange juice (OJP and OJWP) with 2.5, 5, and 7.5 RMD%. (**b**) Mean values and standard deviation of total carotenoids variation of pasteurized orange juice (OJP and OJWP) with 2.5, 5, and 7.5 RMD%. Letters indicate homogeneous groups established by the ANOVA (*p* < 0.05) for each parameter analyzed. OJP, orange juice with pulp; OJWP, orange juice without pulp.

Figure 1a shows that higher concentrations of RMD added before orange juice pasteurization led to a higher (*p* < 0.05) protective effect on TP content in both OJP and OJWP samples. Besides this, OJP samples showed significantly (*p* < 0.05) higher TP variation values than OJWP samples, following the same trend as in control samples (Table 2). The difference between OJP and OJWP samples in terms of TP variations was higher, as higher concentrations of RMD were applied. Therefore, according to several studies, the protective effect that RMD displays on TP might help to preserve, or even improve, the health-promoting and prebiotic-like effect that phenolic compounds exert [18,46,53,54].

The assessment of carotenoids in citrus products is difficult because of their complex carotenoid profile and because of the inherent acidity of these products [55,56]. TC were measured to compare the effect of RMD addition in OJP and OJWP samples. As it was observed in Table 2, OJP0 had significant higher TC content than OJWP0 (*p* < 0.05), probably because the TC content is higher in the pulp than in the orange juice. Figure 1b shows that both OJP and OJWP presented higher positive variations of TC content as RMD concentration increased significantly (*p* < 0.05), an effect greater in OJWP samples.

**Figure 2:**
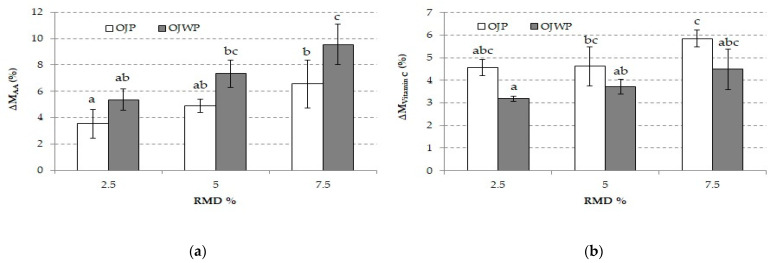
(**a**) Mean values and standard deviation of ascorbic acid variation of pasteurized orange juice (OJP and OJWP) with 2.5, 5, and 7.5 RMD%; (**b**) Mean values and standard deviation of vitamin C variation of pasteurized orange juice (OJP and OJWP) with 2.5, 5, and 7.5 RMD%. Letters indicate homogeneous groups established by the ANOVA (*p* < 0.05) for each parameter analyzed. OJP, orange juice with pulp; OJWP, orange juice without pulp.

Vitamin C comprises two biologically active forms, AA and DHAA [57]. Content variations of AA and vitamin C because of RMD addition in OJP and OJWP samples are represented in Figure 2. Higher RMD concentrations led to significantly increased AA and vitamin C content (*p* < 0.05), more noticeable in OJWP and OJP samples, respectively. Therefore, orange pulp seems to interact with RMD to increase vitamin C protection from oxidative enzymes (i.e., AA oxidase and peroxidase) when the food matrix is disrupted by heat treatment [58]. Loss of vitamin C, because of heat treatment, is undesirable because of its many health-related implications. In fact, the role that vitamin C might play for the prevention and treatment of COVID-19 is being investigated [59]. RMD addition to orange juice has been shown to exert a protective effect on vitamin C, which is of prime interest nowadays. In accordance with these results, Alves Filho et al. [60] pointed out that adding prebiotic fibers, such as inulin and gluco-oligosaccharides, exhibited a protective effect on the vitamin C of heat-treated acerola juice. Finally, despite the observation that RMD addition to orange juice displayed a protective effect on AA and vitamin C content, this effect was more noticeable in TP and TC, as the positive variations registered in those compounds were greater.

**Figure 3:**
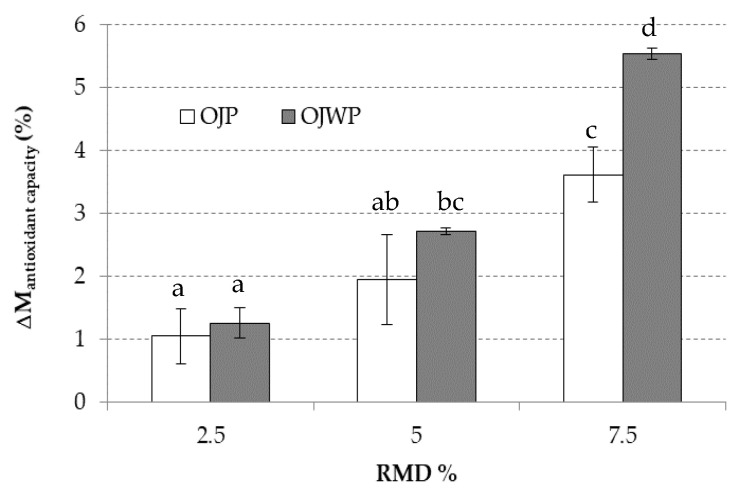
Mean values and standard deviation of antioxidant capacity (AC) variation of pasteurized orange juice (OJP and OJWP) with 2.5, 5, and 7.5 RMD%. Letters indicate homogeneous groups established by the ANOVA (*p* < 0.05) for each parameter analyzed. OJP, orange juice with pulp; OJWP, orange juice without pulp.

Figure 3 shows the AC variation in each RMD-added OJP and OJWP sample. As with all bioactive compounds, the higher the RMD concentration in the pasteurized orange juice, the significantly greater the protective effect on the AC (*p* < 0.05). This was seen in the pulp-free samples, where OJWP7.5 achieved the highest (*p* < 0.05) AC variation. Therefore, adding prebiotic fibers, such as RMD, could play a key role in preserving intrinsic health-promoting compounds from thermal degradation. Fonteles et al. [61] also reported that inulin addition to acerola juice displayed a protective effect on preserving bioactive compounds (vitamin C and TP) after thermal processing, therefore leading to higher AC.

To explain the relationships in the different compounds quantified in this study with the AC and the relationships among them, correlation statistical analyses were performed. The studied bioactive compounds showed a positive Pearson’s correlation coefficient with AC. Vitamin C and TP played a key role in the AC of orange juices, showing 0.8916 (*p* < 0.05) and 0.8647 (*p* < 0.05), respectively. This behavior has been observed by other authors in citric products [17,29,31]. In fruit juices, it is widely accepted that AC is mainly related to AA and TP content [45]. Likewise, Igual et al. [29] found a significant correlation (0.8313, *p* < 0.05) between the AA and TC content in other citric-based products, probably because of the stabilizing effect of AA on carotenoids [62].

### 3.2. Effect of RMD on In Vitro Digestibility and Bioactive Compounds Bioaccessibility of Pasteurized Orange Juice

**Figure 4:**
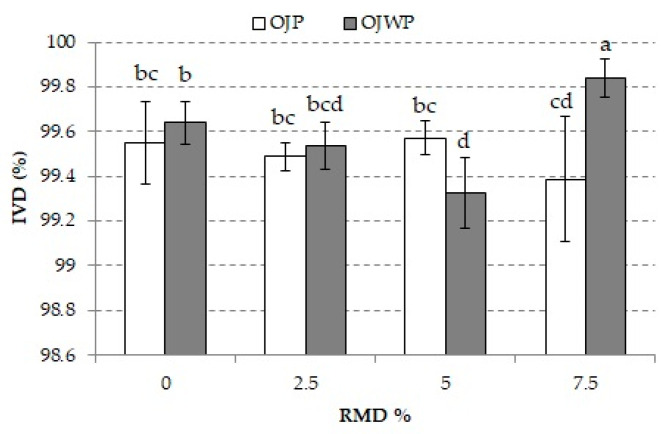
Mean values and standard deviation of IVD percentage of pasteurized orange juice (OJP and OJWP) with 0, 2.5, 5, and 7.5 RMD%. Letters indicate homogeneous groups established by the ANOVA (*p* < 0.05). OJP, orange juice with pulp; OJWP, orange juice without pulp.

Figure 4 shows the IVD% of all OJP and OJWP samples. All orange juice samples obtained a high IVD% because orange juice has an aqueous matrix with monosaccharides (glucose and fructose) and disaccharides (sucrose) [37], which are easy to digest. Although RMD addition to orange juice slightly changed its digestibility, especially when higher doses of RMD were applied, no clear trend was observed. Thus, OJWP5 had the significantly lowest IVD% (*p* < 0.05), whereas OJWP7.5 had a significantly higher digestibility (*p* < 0.05). Moreover, despite not exerting a significant effect (*p* > 0.05), orange pulp slightly reduced the digestibility of the orange juice, because OJP samples presented lower IVD% than OJWP. This could be explained by the fact that orange pulp is an insoluble fiber that typically contains large amounts of cell wall polysaccharides [63], which could pose difficult digestibility compared to the pulp-free orange juices.

**Figure 5:**
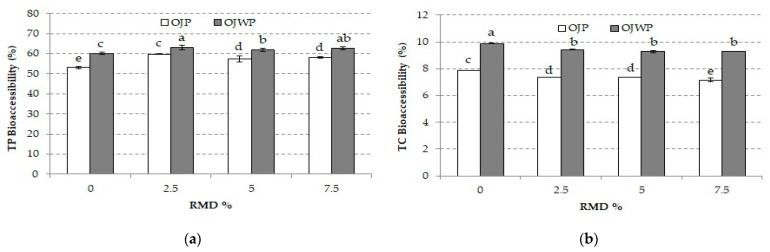
(**a**) Mean values and standard deviation of total phenols (TP) bioaccessibility of pasteurized orange juice (OJP and OJWP) with 0, 2.5, 5, and 7.5 RMD%. (**b**) Mean values and standard deviation of total carotenoids (TC) bioaccessibility of pasteurized orange juice (OJP and OJWP) with 0, 2.5, 5, and 7.5 RMD%. Letters indicate homogeneous groups established by the ANOVA (*p* < 0.05) for each parameter analyzed. OJP, orange juice with pulp; OJWP, orange juice without pulp.

In vitro digestion is also useful for the estimation of pre-absorptive events, such as bioaccessibility of nutrients from a food matrix [64]. Figure 5a shows the TP bioaccessibility of all OJP and OJWP samples. TP bioaccessibility of OJWP samples was slightly higher (*p* < 0.05) than OJP samples, suggesting that orange pulp, despite adding hesperidin to orange juice [46], decreases phenols release from food matrices. However, RMD in the orange juice improved (*p* < 0.05) TP bioaccessibility, although higher RMD concentrations did not have a significant effect (*p* > 0.05). Likewise, Fonteles et al. [61] reported that inulin addition to acerola juice increased TP bioaccessibility. Furthermore, Moser et al. [65] also concluded that adding orange pomace and commercial pulverized citrus pulp fiber to orange juice enhances flavonoids bioaccessibility. In contrast, other studies on fruit juices have reported that the interaction between phenols and dietary fibers in the in vitro digestion process might reduce its solubility and availability [47,58]. The effect of dietary fibers on TP bioaccessibility was more related to the solubilization capacity of the fiber in the food matrix, as orange pulp (insoluble) reduced TP bioaccessibility, but RMD (soluble) presence improved it. In this regard, Schallow et al. [63], who studied the water-binding and gelling properties of orange by-products, suggested that at a low TSS content, more water might be bound by the insoluble pulp fiber, promoting the aggregation of adjacent pectin chains and helping to form larger and more resistant rupture junction zones. Therefore, orange pulp presence could impair TP release from an orange juice matrix. Furthermore, the TP bioaccessibility of all samples was higher than those reported in other blended fruit juice-based (orange, kiwi, and pineapple) [58] and other orange fruit and juice-based [65] studies. Besides this, the pasteurization process has been reported to improve TP bioaccessibility in fruit juices [66,67].

The TC bioaccessibility obtained for all samples (Figure 5b) were in the same range as those reported in other fruit juice-based studies [50,58]. As with TP, orange pulp addition to orange juice also decreased TC bioaccessibility significantly (*p* < 0.05). These results agree with those reported in both in vitro [58] and ex vivo [68] studies that suggested that the bioaccessibility and absorption rate of carotenoids is negatively affected by fiber presence. In fact, intrinsic citrus pectin has been suggested to have a strong inhibitory effect on β-carotene absorption [52,69]. This might also explain why RMD addition to orange juice moderately decreased TC bioaccessibility compared to control samples but significantly (*p* < 0.05), although higher concentrations did not have a stronger effect (*p* > 0.05).

**Figure 6:**
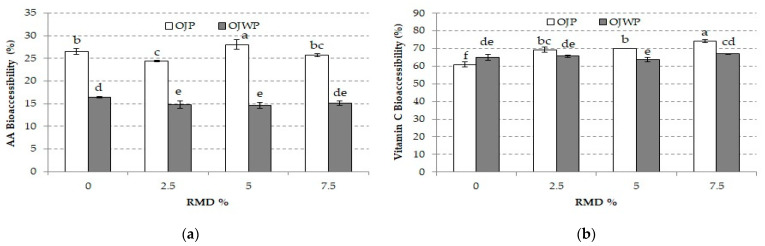
(**a**) Mean values and standard deviation of ascorbic acid (AA) bioaccessibility of pasteurized orange juice (OJP and OJWP) with 0, 2.5, 5, and 7.5 RMD%. (**b**) Mean values and standard deviation of vitamin C (VC) bioaccessibility of pasteurized orange juice (OJP and OJWP) with 0, 2.5, 5, and 7.5 RMD%. Letters indicate homogeneous groups established by the ANOVA (*p* < 0.05) for each parameter analyzed. OJP, orange juice with pulp; OJWP, orange juice without pulp.

Figure 6 shows the mean values and standard deviations of AA and vitamin C bioaccessibility of all OJP and OJWP samples. OJP samples obtained a greater (*p* < 0.05) AA bioaccessibility than OJWP. This indicates that orange pulp might play a protective role in the AA oxidation to DHAA during the in vitro digestion process. RMD addition to orange juice slightly decreased AA bioaccessibility in OJWP samples, although a higher RMD concentration did not have a stronger effect (*p* > 0.05).

Regarding vitamin C bioaccessibility, except for the control samples where OJWP0 obtained a significantly higher vitamin C bioaccessibility (*p* < 0.05) than OJP0, the rest of RMD-added OJP samples showed significantly higher vitamin C bioaccessibility (*p* < 0.05) than OJWP samples. This suggests that RMD incorporation to orange juice might interact with orange pulp to increase DHAA protection during in vitro digestion. RMD significantly improved vitamin C bioaccessibility in OJP samples (*p* < 0.05), especially when higher RMD concentrations were applied, but did not have a significant effect in OJWP samples (*p* > 0.05). Similarly, inulin has been reported to improve vitamin C bioaccessibility in acerola juice [61]. All OJP and OJWP showed comparable vitamin C bioaccessibility to those reported by De Ancos et al. [47], who studied the influence of orange cultivars and mandarins post-harvest on vitamin C during in vitro digestion. Instead, other studies on fruit-based beverages reported lower vitamin C bioaccessibility [58,70,71].

**Figure 7:**
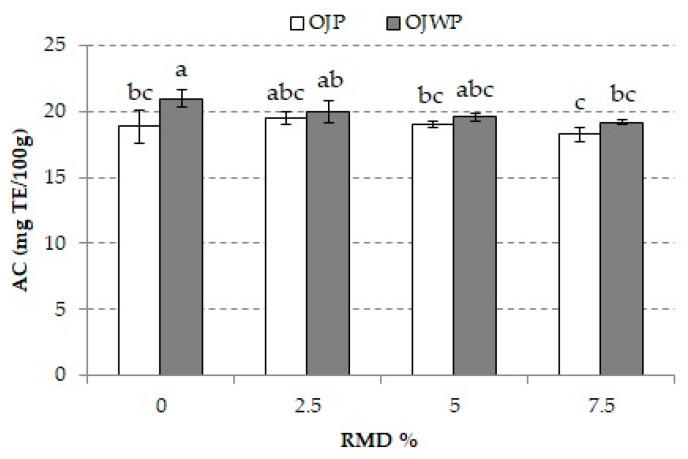
Mean values and standard deviation of antioxidant capacity (AC) of pasteurized orange juice (OJP and OJWP) with 0, 2.5, 5, and 7.5 RMD% after gastrointestinal digestion. Letters indicate homogeneous groups established by the ANOVA (*p* < 0.05) for each parameter analyzed. OJP, orange juice with pulp; OJWP, orange juice without pulp.

Figure 7 shows the mean values and standard deviations of AC of all OJP and OJWP samples after gastrointestinal digestion. The AC ranged from 19.0 ± 1.2 to 21.0 ± 0.7. The OJWP control gave the significantly highest (*p* < 0.05) AC. The OJP control presented significantly lower AC values (*p* < 0.05), probably due to the pulp effect on bioactive bioaccessibility. RMD addition slightly decreased the AC, more noticeably as higher RMD concentrations were added. Consequently, both 7.5% RMD-added OJP and OJWP samples gave the lowest significant AC (*p* < 0.05).

However, even though RMD addition to orange juice displayed a slight effect by diminishing all bioactive compounds’ bioaccessibility, the total amount of bioactive compounds that remained available to be absorbed in the human gut were higher than in control samples. This is because RMD-added samples before in vitro digestion showed a significantly higher bioactive compounds content, in absolute terms, than the control samples.

## 4. Conclusions

RMD addition before the pasteurization juice process protected all bioactive compounds, namely TP, TC, AA, and vitamin C, as well as the AC. Moreover, this protective effect for the bioactive compounds of orange juice was higher when higher RMD concentrations were applied.

Orange pasteurized juice with pulp presented significantly higher values of all bioactive compounds than orange pasteurized juice without pulp; therefore, orange pulp could play a key role regarding the antioxidant properties of pasteurized orange juice.

Concerning bioaccessibility, RMD addition improved TP and vitamin C bioaccessibility but decreased TC and AA bioaccessibility. Subsequently, the AC value of samples after gastrointestinal digestion was slightly decreased by RMD addition. Besides, orange pulp presence decreased TP and TC bioaccessibility but increased AA and vitamin C bioaccessibility.

This study shows that RMD could have interesting applications in the food technology field, leading to health-related benefits. Besides the prebiotic component of RMD, it also displays other important activity to protect health-promoting compounds from degradation because of heat treatment, which is the most common means to preserve fruit juices. However, it would be interesting to know the evolution and stability of the bioactive compounds studied during storage. For this reason, the authors are developing an experiment, and the results obtained now are favorable.

## Figures and Tables

**Table 1:** Mean values (and standard deviations) of TSS, pH, and acidity of pasteurized orange juice.

Sample	TSS	Acidity (g CA/100 mL)	pH
OJP0	11.38 ± 0.03 ^a^	0.77 ± 0.04 ^h^	3.68 ± 0.02 ^a^
OJP2.5	13.58 ± 0.02 ^c^	0.747 ± 0.002 ^g^	3.68 ± 0.02 ^a^
OJP5	15.75 ± 0.04 ^e^	0.725 ± 0.002 ^f^	3.69 ± 0.02 ^a^
OJP7.5	17.99 ± 0.04 ^g^	0.711 ± 0.002 ^e^	3.71 ± 0.02 ^a^
OJWP0	11.47 ± 0.08 ^b^	0.691 ± 0.002 ^d^	3.80 ± 0.03 ^b^
OJWP2.5	13.72 ± 0.03 ^d^	0.670 ± 0.003 ^c^	3.90 ± 0.04 ^b^
OJWP5	15.90 ± 0.05 ^f^	0.6530 ± 0.0005 ^b^	3.83 ± 0.02 ^b^
OJWP7.5	18.09 ± 0.02 ^h^	0.636 ± 0.002 ^a^	3.82 ± 0.02 ^b^

The same letter in superscript within the column indicates homogeneous groups established by ANOVA (*p <* 0.05). OJP, orange juice with pulp; OJWP, orange juice without pulp.

**Table 2:** Mean values (and standard deviations) of total phenols (TP), total carotenoids (TC), ascorbic acid (AA), and vitamin C content of pasteurized orange juice with and without RMD addition, OJP0 and OJWP0, respectively (control samples).

Compounds	OJP0	OJWP0
TP (mg_GAE_/100 mL)	99.8 ± 1.2 ^a^	88.9 ± 0.6 ^b^
TC (mg_β-carotene_/100 mL)	7.13 ± 0.02 ^a^	6.97 ± 0.02 ^b^
AA (mg_AA_/100 mL)	5.81 ± 0.13 ^a^	5.76 ± 0.07 ^a^
Vitamin C (mg_Vitamin C_/100 mL)	6.80 ± 0.04 ^a^	6.66 ± 0.02 ^b^
AC (mg_TE_/100 mL)	106.1 ± 0.5 ^a^	102.3 ± 0.3 ^b^

The same letter in superscript within the row indicates homogeneous groups established by ANOVA (*p <* 0.05).
